# Patient medical costs for tuberculosis treatment and impact on adherence in China: a systematic review

**DOI:** 10.1186/1471-2458-11-393

**Published:** 2011-05-26

**Authors:** Qian Long, Helen Smith, Tuohong Zhang, Shenglan Tang, Paul Garner

**Affiliations:** 1 School of Public Health, Chongqing Medical University, Chongqing, China; 2 Department of Public Health, University of Helsinki, Helsinki, Finland; 3 Liverpool School of Tropical Medicine, Liverpool, UK; 4 School of Public Health, Peking University, Beijing, China; 5 World Health Organization, Geneva, Switzerland

## Abstract

**Background:**

Charging for tuberculosis (TB) treatment could reduce completion rates, particularly in the poor. We identified and synthesised studies that measure costs of TB treatment, estimates of adherence and the potential impact of charging on treatment completion in China.

**Methods:**

Inclusion criteria were primary research studies, including surveys and studies using qualitative methods, conducted in mainland China. We searched MEDLINE, PUBMED, EMBASE, Science Direct, HEED, CNKI to June 2010; and web pages of relevant Chinese and international organisations. Cost estimates were extracted, transformed, and expressed in absolute values and as a percentage of household income.

**Results:**

Low income patients, defined at household or district level, pay a total of US$ 149 to 724 (RMB 1241 to 5228) for medical costs for a treatment course; as a percentage of annual household income, estimates range from 42% to 119%. One national survey showed 73% of TB patients at the time of the survey had interrupted or suspended treatment, and estimates from 9 smaller more recent studies showed that the proportion of patients at the time of the survey who had run out of drugs or were not taking them ranged from 3 to 25%. Synthesis of surveys and qualitative research indicate that cost is the most cited reason for default.

**Conclusions:**

Despite a policy of free drug treatment for TB in China, health services charge all income groups, and costs are high. Adherence measured in cross sectional surveys is often low, and the cumulative failure to adhere is likely to be much higher. These findings may be relevant to those concerned with the development and spread of multi-drug resistant TB. New strategies need to take this into account and ensure patient adherence.

## Background

In China, over 130,000 people die from tuberculosis (TB) each year [1]. In 2004, 140,000 people were estimated to have multidrug resistant tuberculosis (MDR TB), about one third of the total worldwide [2]. It is therefore particularly important that the health system ensures the delivery of full treatment to those with the disease.

China's health system was decentralised in the 1980's, and the central government budget for health facilities dropped to 10% of the total facilities' revenue by the early 1990s [3]. Health services were expected to generate revenue and manage surpluses [3]. Whilst government controlled the price for basic health care, they also established a margin for drug sales so facilities could generate income and survive [4]. These initial reforms meant TB patients were charged like everyone else.

With increased TB incidence in the 1990's thought to be related to rural-urban migration [5], the government responded by abolishing fees for TB diagnosis and treatment. The TB control institutes are authorised to provide free TB drugs (6 months for new patients, 8 months if previously treated); and free X-ray examination at first visit, followed by a free sputum smear test. Accompanying this, health workers in general health facilities are obliged to refer TB suspects and patients to local TB dispensary for diagnosis confirmation and treatment. This national programme was organised centrally and conducted at county (district/city) level; international agencies (e.g. World Bank) helped with subsidies, loans and technical advice [6]. Performance appeared remarkably high, with one report of a defaulter rate of 1.6% in 55,213 new patients [7].

Despite the central government commitment to free treatment, providers have found ways of generating revenue from TB patients. There is clear evidence, for example, that providers recommend treatment beyond the period of free drugs; request repeated investigation and follow up X-rays and blood tests; and prescribe liver protection and ancillary drugs [1,8,9]. All of these require payment, and are added on to the basic package of free care provided in the TB programme. Does this impact on adherence? Although the aggregate national statistics indicate very high completion rates, the programme is heavily target driven. It is possible that the official figures are perhaps optimistic estimates; they certainly hide regional and local variation where performance may be poorer. When carefully conducted, usually small, independent surveys are carried out that record a problem with adherence, there is a difficulty in interpretation: is this just a local problem, or more generalised? In such circumstances, a systematic review of the small studies can help examine this. Given the serious potential public health impact of incomplete TB treatment in 1,500,000 people under treatment each year, we sought to summarise reliable evidence relevant to these debates. We sought to answer three main questions:

1) How much do TB patients pay for medical care?

2) What is the range of estimates for default?

3) Is there any evidence of a link between medical care costs and adherence?

## Methods

### Criteria for considering studies for this review

This review included original research conducted in mainland China, with explicit methods so it was clear how the data had been collected. Criteria were specific to each question: for patient costs and completion rates, we sought surveys; for exploration of links between costs and adherence, we sought relevant surveys and qualitative data.

Inclusion criteria were carefully defined (Table 1) and applied. Studies where there was no information on the study design, study population and how data were collected were excluded. This excludes routine health information collected and collated by the health service. We included research in Chinese or English published between 1979 and June 2010.

**Table 1:** Inclusion criteria

Study question	Inclusion criteria	Data to be sought and extracted
1. Medical costs to TB patient	Population-based surveys or facility-based surveys on TB patients (including new and re-treatment patients)	Total and average cost; cost as a proportion of annual household income
2. Adherence rates	Population based surveys or cohort studies	Completion or cure per patient followed up
3. Impact on completion and adherence	*Surveys *must be population based, with household or individual cost estimates from interviews and completion rates based on verified health service data, or on patient reported length of treatment	A proportion of interrupted treatment because of financial burden caused by TB treatment
	*Qualitative studies *that a) use recognised methods (in-depth interviews, focus group discussions, or observation); and b) describe the methods used in analysis (thematic analysis, content analysis, grounded theory)	Patient or provider views on cost and its influence on treatment completion. Include evidence, in the form of illustrative quotes or empirical data, for the statements

### Search strategy

We searched the following databases: MEDLINE, PUBMED, EMBASE, Science Direct, HEED with the following search terms: Tuberculosis, adherence, treatment, completion, cure; cost, financial burden; the poor, low income, vulnerable group, migrants; Directly Observed Treatment short-course (DOTs), initiatives, incentive, health insurance, New Cooperative Medical Scheme, China. The electronic search strategy for one database is in Table 2. One Chinese database (CNKI) was searched using terms "tuberculosis, treatment, cost, financial burden, adherence". This search was to identify studies in Chinese that were not included in English databases presented above. Google search and web pages of TB control of Chinese Ministry of Health and other international organisations (including the World Health Organization, World Bank) were screened in October 2010 and April 2011 for further relevant studies by searching on "(tuberculosis or TB control) and "China".

**Table 2:** Electronic search strategy for one database (Medline)

1 China [Mesh], ti, ab
2 Tuberculosis [Mesh], ti, ab
3 1 AND 2
4 Patient compliance [Mesh]
5 adherence OR DOT* OR (directly observed) ti, ab
6 (treatment completion) OR cost* OR finance* OR vulnerable OR incentive* OR insurance OR fees OR monetary OR survey*) ti, ab
7 (low income*) OR (vulnerable group*) OR migrant* ti, ab
8 4 OR 5 OR 6 OR 7
9 3 AND 8

### Data extraction and transformation

The first author identified studies based on examining titles, and then abstracts. Full texts of relevant abstracts were retrieved for further assessment. Data were extracted using a standard form. Uncertainties were resolved through discussion with co-authors and contact with study authors. Data were extracted onto a single form and the data extraction was checked by a second author. The main outcome we sought was medical cost of treatment; the variation in measuring cost of TB treatment required some data conversion, that we did using standard assumptions about length of treatment for six months. Furthermore, we extracted or calculated cost as a percentage of annual household income as an indicator to assess TB patient's financial burden by different study areas. We summarised data as brief narrative for outcome measure.

## Results

### Description of studies

**Figure 1:**
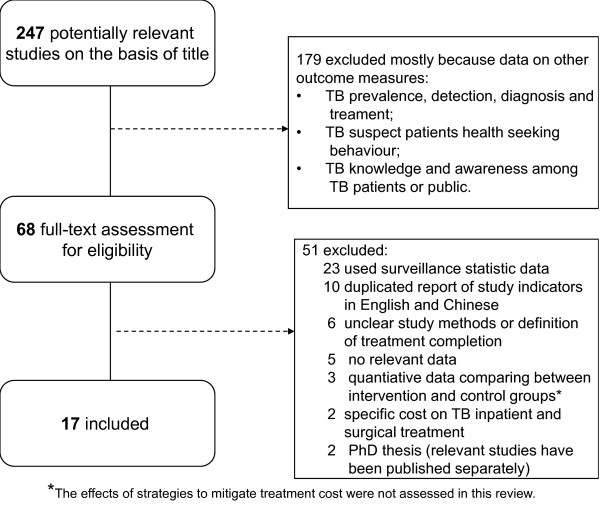
Identification of studiesD

Figure 1 presents the review profile. The search yielded 1143 studies for TB related diagnosis and treatment, of which 247 studies (including 12 studies having qualitative study design) potentially met the inclusion criteria. On the second screening, we excluded studies on the basis of the abstract. Sixty-eight studies were retrieved for full-text evaluation after screening the abstracts. Fifty-one studies were excluded because they reported routine health service surveillance data, or because they were duplicate publications of a study already included, they provided no relevant data, or no study methods or the definition of treatment completion was not clear (Figure 1). Of the four national TB epidemic surveys between 1979 and 2000, only the 2000 study reported treatment completion rate and was included.

In all, 17 studies (14 in English and 3 in Chinese) met the inclusion criteria for questions 1-3. The study design included surveys (14), both quantitative and qualitative methods (2), and qualitative design only (1). Studies were national (2); limited to 4 selected provinces in all regions (1); eastern (7); western (4); and central (2); and unknown region due to authors concerns about confidentiality (1). Most counties studied were middle or low income; eleven studies were concerned wholly with counties designated as World Bank/TB Programme areas where TB drugs were supplied for free (Table 3).

**Table 3:** Description of included studies

Author year	Data collected	TB patient (n)	Province	Sample unit	Income category, status of free treatment	Methods	Outcomes	Data collection	**Questions **^**a**^		
									1	2	3
Liu 2010^8^	2007	163	Chongqing Sichuan	4 counties in each province	8 mixed income, All free	Patient survey	Reported cost in previous month on TB drugs and tests	Postgraduate students	●		
Zhan 2004^9^	2000-2001	257	Shandong Jiangsu	Counties	3 middle, 1 free 2 not free	Patient survey	Reported total medical cost per treatment	Health staff	●		
Meng 2004^10^	2000	312	Shandong	Counties	2 middle income, 2 poor: All free	Patient survey	Reported total medical annual cost per TB patient	Medical students and health staff	●		
Liu 2007^14^	2004	889	Fujian Henan Liaoning Xinjiang	3 counties in each province	12 poor, All free	Patient survey	Reported direct household cost per treatment	Health staff from non-TB system and university students	●		
Huang 2009^16^	2007	100	Hunan	Counties	10 unknown, All free	Patient survey	Reported total medical cost per treatment	Unknown	●		
Xu 2006^15^	2002-2003	465	Jiangsu	Counties	2 middle income, 1 free 1 not free	Longitudinal: 4 interviews per patient ^b^	1. Reported total medical cost for treatment 2. Non-adherence rate	TB specialist	●	●	
Xu 2010^13^	2008	501	Shandong	Counties	6 mixed income, All free	Patient survey	1. Reported annual household medical cost in household with TB patient 2. Non-adherence rate	Independent investigator with help of TB staff	●	●	
Zhang 2007^12^	2002	182	Whole of rural China	N-A	N-A	Patient survey	1. Reported annual household medical cost in household with TB patient 2. Influence of charging on treatment	Township health workers	●		●
Jackson 2006^11^	2002-2005	160	Henan	Counties	4 mixed income, All free	Longitudinal: 2 interviews per patient ^c^	1. Reported total medical cost per completed treatment 2. Non-adherence rate 3. Influence of charging on treatment	TB specialist	●	●	●
Wang 2007^18^	2006	130	Yunnan	Counties	10 mixed income, All free	Patient survey	Non-adherence rate	Village doctors and local CDC staff		●	
Wang 2009^21^	2004-2007	607	unknown	Counties	2 high income, All free	Patient survey	Non-adherence rate	Independent investigator		●	
NESS-TB 2002^17d^	2000	1278 ^e^	Whole of China	N-A	N-A	Patient survey	1. Non-adherence rate 2. Influence of charging on adherence	unknown		●	●
Hu 2008^19^	2004	405	Chongqing	Counties	4 mixed income, 2 free 2 not free	1. Patient survey 2. In-depth interview	1. Non-adherence rate 2. Influence of charging on adherence	Postgraduate students		●	●
Xu 2009^20^	2006	670	Jiangsu	Counties	13 mixed income, All free	1. Patient survey 2. In-depth interview	1. Non-adherence rate 2. Influence of charging on adherence	TB staff		●	●
Ai 2010^22^	2006-2007	659	Shaanxi	Counties	30 poor, All free	Patient survey	1. Non-adherence rate 2. Influence of charging on adherence	Unknown		●	●
Sun 2007^23^	2004-2005	473	Jiangsu	County	1 unknown, Free	Longitudinal: 3 interviews at 2^nd^, 5^th ^treatment month and treatment completion	1. Non-adherence rate 2. Influence of charging on adherence	TB staff		●	●
Xu 2004^24^	unknown	30	Jiangsu	Counties	2 middle income, 1 free 1 not free	Focus Group Discussions (FGDs)	Influence of charging on adherence	Researcher and postgraduate students			●

N-A: not applicable

^a ^In the column "Questions", point refer to which question the paper contributes data to

^b ^Good follow up (>92% complete all interviews);

^c ^After 10-12 months 11 people died and five could not be located.

^d ^NESS-TB: National technical steering group of the epidemiological sampling survey for tuberculosis

^e ^900 new cases and 378 patients who received treatment

### Medical costs

Nine studies provided estimates of total medical costs per treatment (excluding transport and food) (Liu 2010, Meng 2004, Jackson 2006, Zhang 2007, Xu 2010, Liu 2007, Xu 2006, Zhan 2004 and Huang 2009) (Table 4):

**Table 4:** Annual medical costs for one TB treatment (RMB). Excludes costs for diagnosis

Study	Methods	n	Variables	County or patient group	TB treatment	Annual cost	% of annual household income
Liu 2010^8^	Method: Survey Includes: All TB patients Extent: 8 counties	163	Average expenditure in previous month on TB drugs and tests	Income below poverty line <3720/year (n = 24)	Free	1584 ^a^	93%
Meng 2004^10^	Method: Survey Includes: All patients Extent: 4 counties	312	Average total medical annual cost per patient	Poor county 1 (n = 81) Poor county 2 (n = 65)	Free Free	3070 2241	119% 57%
Jackson 2006^11^	Method: Case control Costs prospective Includes: All patients Extent: 4 counties	160	Direct medical costs per completed treatment	Mixed income (n = 144)	Free	1940	39% ^b^
Zhang 2007^12^	Method: Household survey Number: 143,991 people Includes: TB patients Extent: National	180	Annual household medical cost	Low income (n = 80)	Mixed	1241	42%
Xu 2010^13^	Method: Survey Includes: All TB patients Extent: 6 counties	501	Annual household medical cost	Poor county 1 (n = 68) Poor county 2 (n = 88)	Free Free	5228 4079	65% 84%
Liu 2007^14^	Method: Survey Includes: Random sample (TB patients) Extent: 3 poor counties in each of 4 provinces	889	Direct medical cost per treatment	Province 1 (n = 217)) Province 2 (n = 228) Province 3 (n = 223) Province 4 (n = 221)	Free Free Free Free	775 800 1760 445	8% ^c ^ 11% 27% 5%
Xu 2006^15^	Method: Cohort study Includes: All TB patients Extent: 2 counties	465	Direct medical cost per treatment	Middle county 1 (n = 183) Middle county 2 (n = 282)	Free Charged	90 704	2% ^d ^ 12%
Zhan 2004^9e^	Method: Survey Includes: All patients^f ^Extent: 3 counties	257	Direct medical costs per treatment	Middle county 1 (n = 46) Middle county 2 (n = 105)	Free Charged	1517 1754	NA
Huang 2009^16^	Method: Survey Includes: Sample (TB patients) Extent: 10 counties	100	Direct medical costs per treatment	Unknown	Free	1635	NA

NA: not available

^a ^Monthly costs estimated multiplied by six; Monthly household income reported multiplied by twelve

^b ^From reported average annual household income of the TB patients interviewed

^c ^From total cost as a percentage of annual household expenditure

^d ^Based on reported average annual household income of the TB patients interviewed

^e ^Cost in US$ converted to RMB by exchange rate used in the study. One county excluded as data from health worker estimates

^f ^Random sample in one of the three counties

• RMB 1584 (US$ 220) per household for one treatment was the average medical cost per treatment among low income groups in 8 counties surveyed in a study (n = 24), representing 93% of the total annual household income in this group [8].

• RMB 2241 to 3070 (US$ 270 to 371) per household for one treatment was the range for the average cost in each of 2 poor counties surveyed in a study (n = 146), representing 57% to the maximum of 119% of average annual household income [10].

• RMB 1940 (US$ 242) was the total average out-of-pocket payment for medical costs from diagnosis to completion representing 39% of annual household income in one carefully conducted study. The authors interviewed the same patients (n = 144) on two occasions in 4 counties where drugs were supplied for free [11].

• RMB 1241 (US$ 149) of annual household medical expense among low income households with a TB patient (as a proxy indicator to estimate medical cost for TB treatment) was the finding in the fourth study (n = 80), representing 42% of annual household income in this group [12].

• RMB 4079 to 5228 (US$ 565 to 724) was the range for annual household medical expense in two poor counties (as a proxy indicator to reflect financial situation of TB patients) in a fifth study (n = 156), representing 65% to 84% of average annual household income [13].

• RMB 445 to 1760 (US$ 54 to214) was the range for the average medical cost per treatment in each of 3 poor counties of 4 provinces in a sixth study, representing 5% to 27% of annual household expenditure [14].

• RMB 90 to 1754 (US$10 to 213) was the direct medical cost per treatment by county with or without free treatment in two studies [9,15].

• RMB 1635 (US$ 199) was the average direct medical cost from diagnosis to treatment completion in 10 counties with free treatment in one study [16].

**Relationships with cost**: In 3 studies (Liu 2010, Meng 2004 and Zhang 2007) [8,10,12] charges were disaggregated by household income, there was no obvious relationship between charges and the income category of the household; and in Meng, between poor and middle income counties (Table 5). However, the average cost as a proportion of total household income increased in the low income groups.

**Table 5:** Estimates of charging: medical cost for TB care by income category (RMB)

Study	Cost variable	County	**Income category **^**a**^			Average cost, % annual household income
				
			Low	Middle	High	
Meng 2004^10^	Total medical cost	Middle	2099	2826	3105	75%
		Middle	2104	1449	1680	27%
		Poor	4015	2011	3238	119%
		Poor	1843	2054	2894	57%
Zhang 2007^12^	Annual household medical cost in household with TB patient	Whole rural China	1241 (42%)^b^	2061 (28%)^b^	2090 (19%)^b^	NA
Liu 2010^8^	Monthly medical cost	Mixed	264 (185%)^c^	280 (22%)^c^	332 (8%)^c^	18%^c^

NA: not available

^a ^In Meng's study, Income category was classified by per capita income per year (Low≤500; Middle 500-1000; High > 1000)

In Zhang's study, each income category contained a third of the total number of households;

In Liu's study, income category was classified by family monthly income (Low < 310; Middle 310-2600; High > 2600);

^b ^Average annual household medical cost in household with TB patient as a percentage of annual household income in each income group;

^c ^Average monthly medical cost as a percentage of family monthly income in each income group and at an average level.

In two studies (Zhan 2004, Xu 2006) [9,15] comparing counties, the "free treatment" counties had diagnostic costs 2-3 times that the other counties that charge for TB drugs, suggesting transfer of costs from one function of the TB service to another. In one of the two studies (Zhan 2004), drug treatment costs were high despite this being a county with free treatment.

All but one study gave a single figure for "medical costs". The exception was Liu (2010) [8]: the authors asked patients about the cost components, and showed these consisted of prescribing of liver protection, immune stimulant and anti-inflammatory drugs, and monthly X-ray and blood tests, with an average of RMB 287 (US$ 40) charged to each patient in the previous month. In the low income group, cost of liver protection drugs was 71% of patient monthly income; 32% and 63% of patient income being spent on ancillary drugs and tests respectively.

### Adherence to treatment

Ten studies were surveys in which the investigators asked whether people who should have been on treatment were actually taking their drugs (Table 6). The large national sample survey in 2000 showed that 73% of patients underwent interrupted or suspended treatment (39% in TB services system and 79% in general health system) [17].

**Table 6:** Non-adherence estimates from studies

Study	Study design	n	Definition of non-adherence	Non-adherence rate
Xu 2006^15^	Cohort: 4 interviews per patient	465	Did not complete treatment course	5%-9%
Jackson 2006^11^	Cohort: 2 interviews per patient	144	Partial treatment	11%
Sun 2007^23^	Cohort: 3 interviews per patient	473	Interrupted or defaulted treatment	13%
Xu 2010^13^	Patient survey	501	Defaulted and failed treatment	25%
Wang 2007^18^	Patient survey	130	Stopped treatment > 2 weeks or missed over 20% of pack	3% or 4%
Hu 2008^19^	Patient survey	401	Missing last three doses, or no stock >1 week	13%
Xu 2009^20^	Patient survey	670	Missing 10% or more of the total pack	12%
Wang 2009^21^	Patient survey	537	Did not complete treatment course	15%
Ai 2010^22^	Patient survey	659	Defaulted treatment ^a^	12%
National TB epidemiological survey 2002^17^	Patient survey	378	Interrupted or suspended treatment	73%

^a ^82 defaulted from treatment for which 20 died.

In 6 smaller studies (Xu 2010, Wang 2007, Hu 2008, Xu 2009, Wang 2009 and Ai 2010), failure to follow standard treatment course varied from 3% to 25% across these studies [13,18-22]. Although each study used different outcomes, all appeared to measure the point prevalence of non-adherence; this does not provide the cumulative effect over the entire treatment period. Three studies followed up a cohort (Jackson 2006, Xu 2006 and Sun 2007) reported rate of incomplete treatment course from 5% to 13% [11,15,23].

### Association between patient costs and adherence

The national survey and 4 smaller studies examined reasons why patients had interrupted treatment (National TB epidemiological survey 2002, Jackson 2006, Zhang 2007, Xu 2009 and Ai 2010). The percentage of non-adherence for reasons of cost varied from 3% to 45% of the total number of patients (Table 7) [11,12,17,20,22]. One study (Sun, 2007) examined the association between treatment cost and adherence showing higher cost with higher likelihood of non-adherence [23].

**Table 7:** Studies reporting relationship between charging and adherence

Study design	Study	Comment
Survey	National TB epidemiological survey 2002^17^	45% (121/272) of patients having interrupted or suspended treatment due to financial difficulty
	Zhang 2007^12^	9% of households with TB suspects (2308) or patients defaulted due to financial burden
	Xu 2009^20^	16% (13/82) of non-adherence patients interrupted treatment because of high medical costs of the treatment
	Ai 2010^22^	2% of patients who interrupted treatment (43) because of financial difficulty
Cohort	Jackson 2006^11^	3% (5/159) was too poor to begin treatment
	Sun 2007^23^	Patients who were charged high medical cost were more likely to interrupt treatment than patients having lower medical costs

In the three studies that met our inclusion criteria for qualitative designs, a link between costs and adherence was identified in them all. Xu (2004), conducted in a county that did not implement free treatment, reported that treatment interruption occurred frequently due to shortage of money, particularly after the first two months of treatment [24]. Under free treatment, Xu (2009) indicated that patients complained of adverse reactions and low quality of free drugs; and doctors admitted to recommending patients buy drugs rather than taking the free drugs. Both patients and local doctors reported that privately purchased drugs imposed a higher financial burden to TB patients, sometimes resulting in interrupted treatment [20]. In another study (Hu 2008) the high cost of treatment was one of the main reasons mentioned by patients who interrupted treatment and village doctors agreed that financial barriers led to default [19]. In two studies, patients specifically described having to pay extra for examinations and additional drugs (mainly liver protection drugs) related to treatment. Patients reported that these additional costs were significant, accounted for a large part of family income, and were related to treatment adherence [19,20].

## Discussion

### Summary of findings

Overall, low income patients, defined at household or district level, pay large amounts of money for medical treatment of TB, ranging from US$ 149 to 724 (RMB 1241 to 5228); as a percentage of annual household income, estimates range from 42% to 119%. One national survey showed 73% of TB patients at survey had interrupted or suspended treatment, and estimates from 9 smaller more recent studies of showed that the proportion of patients at the time of the survey who had run out of drugs or were not taking them ranged from 3 to 25%. Synthesis of surveys and qualitative research indicate that cost is the most cited reason for default.

### Methodological and other potential limitations

#### Limitations of primary studies

Medical costs of TB treatment were measured in different ways in each study. This includes two studies which used annual household medical expenditure as proxy indicator. Obtaining data on expenditure is complex. In these studies, there is likely to be considerable variation in estimates due to a) the rigor of the questions; b) the parameters of cost estimated; and c) the region, province and locality where the research was carried out, as costs vary considerably in China. Few studies provided disaggregated data of the different components of the additional cost (such as tests, additional drugs, or extending treatment).

#### Limitations of our review

This study reviews population-based studies other than routine register data considering reliability of information, but unpublished studies are not included due to practical constraints. As well as the limitations in the evidence we reviewed, we made assumptions in trying to obtain a uniform measure of cost across studies, which could contribute additional uncertainty to the estimates. Another limitation of our study is that the conclusions are based on indirect evidence and inference of an effect from very varied data from different regions of China: we cannot be clear how far all these findings are generalizable across the country.

### Interpretation

In a primary health care system where fee for service is normal, the Chinese government commendably made TB drugs free. However, charges remain universal. It appears free drugs are overlaid on an existing health system based on fee for service, which proves problematic given the existing organisational norms and culture. The charges arise from additional and often unnecessary drugs and tests beyond those supplied through the national programme [8].Thus the revenue-driven practices in the general health system are influencing TB control, including overprovision, poor referral (to keep patients), and high hospitalisation costs [1,9].

We did identify policies to try and mitigate treatment cost but data were limited. A number of pilot schemes are underway, including decentralisation of TB diagnosis and case management services to the township level [25]; travel subsidies to get to clinic; payment to doctors for directly observing treatment [26]; free treatment for migrant patients [27]; and schemes linked to the New Cooperative Medical Scheme (NCMS), including case based payment.

The NCMS in China since 2003 has reached over 90% of the rural population, and has improved the use of healthcare by insured rural residents [28,29]. However, reimbursement is low, and ambulatory patients with chronic diseases have limited financial protection, and still have to pay considerable amounts out of pocket [30-32].

It seems inevitable that this will impact on completion rates. Using surveys, there is a large variation in estimates of TB completion. The national TB survey in 2000 reported a low adherence rate, particularly low in the general health system. In recent studies reported here, the results are the proportion of patients not taking drugs at the time of the survey: the cumulative failure to complete is likely to be higher. Hence, the adherence on average was not so optimistic. What these data do is generate a degree of uncertainty around true completion rates.

TB treatment completion rate from the independent studies reported in this review contrast with published health service statistics which show very good performance [33]. The Ministry of Health in China and others have very clear targets for detection and treatment, set by government and endorsed by World Health Organization. It may be that the actual health information systems do not operate as well as expected, and where there are gaps in the data, health workers worldwide tend to take the most optimistic standpoint in their estimates. Thus the imputed completion rates may be overly optimistic. This we have found in a study in Chongqing [19], although there has been very little systematic assessment of the extent of this problem.

Given the number of patients in China, poor or irregular adherence could have massive public health implications, potentially increasing the risk of multi-drug resistant TB and extremely drug-resistant TB developing. This will cause a worsening of the TB epidemic in areas of China, and will increase the risk of morbidity and mortality among the poor as well as the costs associated with illness.

## Conclusions

Despite a policy of free drug treatment for TB in China, health services charge all income groups, and costs are high. Adherence measured in cross sectional surveys is often low, and the cumulative failure to adhere is likely to be much higher. It is important that there is an open debate between health providers, the Ministry of Health, the World Health Organization and donors about these realities, and discussion about the public health impact. Policy makers need to consider appropriate steps to document further problems with adherence, charging, and how best to improve TB treatment delivery in poor patients.

## Competing interests

The authors declare that they have no competing interests.

## Authors' contributions

This review was designed collectively. QL retrieved references, applied inclusion criteria and extracted data. PG and HS assessed the eligibility of the retrieved papers, participated in the data analysis, results interpretation and the review writing. ST and TZ advised on data presentation interpretation, and contributed to the manuscript. All authors read and approved the final manuscript.

## Pre-publication history

The pre-publication history for this paper can be accessed here:

http://www.biomedcentral.com/1471-2458/11/393/prepub
